# A Case of Poisoning

**Published:** 1854-12

**Authors:** M. A. Milner

**Affiliations:** Pairfield, Texas


A Case of Poisoning. Reported by M. A. Milner, M. D., of Pairfield, Texas.—The comparatively rare occurrence of poisoning by *Hydrocyanic Acid*, or preparations containing it, as cyanide, or cyanuret of potassium, is admitted; yet it does sometimes occur, and this induces me to report the following case, with the hope that it may elicit further enquiry for a certain antidote, and the proper management of such accidents.

On the 6th of October, Col. W's. little daughter, Fanny, three years old, in company with others, visited a Daguerrian gallery, in the second story of my office. While the artist was preparing a plate, one of the ladies gave the child (for water) a solution of the chloride of silver and cyanide of potassium, used to galvanize plates. The mistake was discovered immediately, and the child brought into my office, with the cry, "do something quick! it is poisoned with the chloride of silver." One glance revealed to me the truth of the alarm. Her face was flushed, her breathing slow and sterterous, and she was apparently insensible. With all possible haste, I tried a mustard emetic, but found she could not swallow, and immediately resorted to the stomach pump, and succeeded in drawing off a good portion of the fluid contents of the stomach. It being a short time after dinner, however, the imperfectly chewed and undigested bits of meat, &c., would fill the eyes of the tube, and prevented as effectual an emptying of the stomach as was desired, I then forced salt water into the stomach, but to no purpose, for the child was *dead.*

The length of time from the drinking of the poison, until the last gasp for life, was between four and five minutes.

None of the antidotes, as laid down by authors, were used, such as chlorine water, ammonia, cold water, &c.

In conclusion, I would respectfully ask, where such prominent poisons are taken, and the paralytic effect on the nervous system so instantaneous, should emetics, or the stomach pump, be thought of for relief? Or should we depend upon inhalations and the administration of antidotes?

The composition of the poison was about ℥iv. cyanide potassium, ℥i. chloride silver, and three pints water.—*South. Med. & Surg. Jour.*

